# Editorial: Neutrophil heterogeneity in healing and tissue damage

**DOI:** 10.3389/fimmu.2024.1537469

**Published:** 2024-12-23

**Authors:** Jorge A. Masso-Silva, Gary L. Bowlin

**Affiliations:** ^1^ Division of Pulmonary, Critical Care, Sleep and Physiology, University of California, San Diego, San Diego, CA, United States; ^2^ Pulmonary Critical Care Section, VA San Diego Healthcare System, San Diego, CA, United States; ^3^ Department of Biomedical Engineering, University of Memphis, Memphis, TN, United States

**Keywords:** neutrophils, heterogeneity, sepsis, inflammation, NETosis, neutrophil diversity

As the most abundant leukocyte in circulation, neutrophils are important in homeostasis and disease. Although early studies on neutrophils did not recognize them as a heterogenous cell population, it is now appreciated the diverse phenotypes and roles that can be found in neutrophils. In the context of inflammation, neutrophils are recognized as key cells in protecting against infections, although, when exacerbated inflammation occurs, these cells can cause significant tissue damage. Therefore, this Research Topic called “*Neutrophil Heterogeneity in Healing and Tissue Damage*” aimed to explore the diversity of neutrophils in different scenarios. Below, we briefly discussed the articles included in this Research Topic.

As key drivers for inflammation, neutrophils have been widely studied in the context of sepsis, which is characterized by exacerbated systemic inflammation leading to organ dysfunction. Sepsis is a highly heterogeneous syndrome due to intrinsic human heterogeneity and the diverse scenarios that can lead to sepsis. Thus, Yang et al. sought to identify distinct functional phenotypes of neutrophils in critically ill ICU sepsis patients using an Organ-on-Chip assay to categorize sepsis patients into distinct phenotypes using patient data, neutrophil functional analysis, and proteomics. They were able to identify three functional phenotypes based on *ex vivo* functional assays (adhesion and migration patterns), Hypoimmune, Hyperimmune, and Hybrid. Importantly, they were able to associate these functional phenotypes with distinct proteomic signatures and disease severity. This study aligns with previous studies categorizing septic patients based on a variety of markers (including IL-8, key for neutrophil recruitment and activation) as hypoinflammatory and hyperinflammatory phenotypes in both sepsis and acute respiratory distress syndrome (ARDS) (1). In addition, the study published in this Research Topic by Willemsen et al. offers promising insights into the fight against abdominal sepsis. As research progresses, we recognize that NETosis has a dual role: it aids in the elimination of pathogens but can also hinder this process by protecting pathogens from phagocytosis and promoting antibiotic resistance, effectively acting as a “neutrophil-derived secondary biofilm”. In a murine model of sepsis, the combination of DNase1 and antibiotics significantly improved the clearance of Gram-negative bacteria, reduced inflammation, and increased survival rates. The study underscores how insufficient clearance of neutrophil extracellular traps (NETs) can exacerbate sepsis severity, particularly with Gram-negative bacteria. By combining DNase1 with antibiotics, this research proposes a novel treatment strategy for sepsis that targets both the pathogens and the harmful immune response generated by the host. Furthermore, another study focused specifically on sepsis-associated ARDS, where they assessed a specific neutrophil population characterized for the expression of CD16^bright^ and CD62L^dim^ previously found to be immunosuppressive (2). Here, Zhang et al. evaluated the changes of this population in patients with sepsis-associated ARDS, where they found that this neutrophil population is elevated in these patients and is associated with poor prognosis, suggesting their use as predictors for future clinical complications. Altogether, this area of study gets us closer to better understanding the status of sepsis and sepsis-associated ARDS in the context of the contribution of neutrophils to illness severity and how we could potentially employ more effective therapies to these patients based on their neutrophil phenotypes, where future clinical studies could investigate whether the combination therapies such as DNase1 together with specific antibiotics could be a breakthrough in the management of sepsis.

Fever and hypothermia represent two opposing physiological responses to systemic inflammation. Fever stimulates immune activation, while hypothermia promotes energy conservation. Both conditions are significant in the context of Systemic Inflammatory Response Syndrome (SIRS), a major cause of mortality worldwide. A crucial component of the body’s defense against infection is the neutrophils, which release NETs to capture and eliminate pathogens. Recent research by Janko et al. examined how temperature affects the kinetics of NET release, which is directly linked to enzyme activity and their degradation. The study discovered that NET formation increased at 40°C; however, responses were reduced at both 35°C and 42°C. Interestingly, the degradation of NETs was enhanced at higher temperatures, likely due to increased activity of plasma DNase. These findings indicate that temperature plays a critical role in regulating the release and degradation of neutrophil NETs. Overall, this research could pave the way for new therapeutics aimed at modulating immune responses in conditions like SIRS, pending further clinical testing and validation.

Without a doubt, the study of neutrophil heterogeneity at a single cell resolution has helped to now recognize neutrophils as a heterogenous population, which was not the case 20 years ago, when NETs were discovered in 2004 (3). The use of single-cell RNA-seq (scRNA-seq) analyses has allowed us to define neutrophil diversity in homeostasis and disease in anatomical compartments where usually neutrophils are not studied in depth. An example is the study performed by Villagómez-Olea et al., on the periodontal ligament, which contains significant cellular heterogeneity, including the presence of neutrophils. Using scRNA-seq, they identified 4 distinct neutrophil populations, and interestingly, all these populations seem to represent different stages of neutrophil undergoing maturation. Hence, authors suggest that the periodontal ligament can serve as a niche where neutrophils can mature, serving to some extent as a place for granulopoiesis. Although extramedullary hematopoiesis is generally a compensatory mechanism in response to bone marrow dysfunction or increased demand for blood cells (mostly during inflammation), it is not a regular site of granulopoiesis in healthy adults (4). Thus, this study may uncover a niche where extramedullary granulopoiesis can occur in homeostatic conditions.

In summary, this Research Topic includes studies addressing neutrophil heterogeneity defined by different methodologies, such as cell surface markers, functionality, and scRNA-seq, providing key insights into different physiological conditions. Nevertheless, there are still many challenges in understanding neutrophil heterogeneity. Although, with the rise of single-cell technologies, we now have the opportunity to better define the heterogeneity of neutrophils in-depth, that in combination with longitudinal studies, we could better understand them under homeostasis and disease over time to define key cellular and molecular changes, aiming for better therapies targeting neutrophils when is most needed.
